# Effects of using a mobile health application on the health conditions of patients with arterial hypertension: A pilot trial in the context of Brazil’s Family Health Strategy

**DOI:** 10.1038/s41598-020-63057-w

**Published:** 2020-04-07

**Authors:** Raquel Debon, Ericles Andrei Bellei, Daiana Biduski, Simiane Salete Volpi, Ana Luisa Sant’Anna Alves, Marilene Rodrigues Portella, Ana Carolina Bertoletti De Marchi

**Affiliations:** 1 Graduate Program in Human Aging, School of Physical Education and Physiotherapy, University of Passo Fundo (UPF), Passo Fundo, RS Brazil; 2 Graduate Program in Applied Computing, Institute of Exact Sciences and Geosciences, University of Passo Fundo (UPF), Passo Fundo, RS Brazil

**Keywords:** Interventional cardiology, Palliative care, Patient education, Public health, Outcomes research

## Abstract

Brazil’s Family Health Strategy (FHS) leads public health policies and actions regarding community health, addressing arterial hypertension (AH) in primary care settings. In this scenario, the use of communication technologies becomes appropriate for the monitoring of patients with AH. To preliminary verify the intervention approach and the effects of using an m-Health application on the health conditions of patients with AH for a future study, we conducted a non-randomized, controlled, non-blind trial (N = 39), comparing the use of a mobile health app (m-Health) with conventional AH monitoring over 3 months. During the study, we promoted health information workshops to engage patients from both intervention and control groups. Pre and post-intervention, we compared measurements of systolic and diastolic blood pressure; food frequency questionnaire; Appraisal of Self-Care Agency Scale; blood tests of hemogram, creatinine, uric acid, sodium, potassium, lipid profile, and glycemia. Improvements were identified in both groups due to the workshops, including the reduction in total and non-HDL cholesterol, healthier consumption of salads and sugary drinks, and increased self-care scores. Exclusively in the intervention group, which used the m-Health app, there was a change in systolic and diastolic pressure towards more adequate levels. In addition, the intervention group had improved levels of glucose and HDL cholesterol and reduced consumption of ultra-processed foods. In conclusion, the use of an m-Health app had positive effects on the health conditions of patients with AH under treatment within FHS, especially when combined with health information. On the context of FHS, the use of technology is encouraging supporting better health conditions.

## Introduction

Arterial hypertension (AH) is one of the main modifiable risk factors for diseases of the circulatory system. With a high prevalence, AH is considered one of the most important public health problems. The World Health Organization estimates that about 600 million people have AH and, annually, 7.1 million individuals die from this disease, substantially burdening health systems^1^. Among the AH modifiable factors, either by medication or by altering habits and behavior, there is obesity, sedentary lifestyle, eating habits, and stress. Controlling blood pressure can still be a challenge since, with everyday activities and lack of time, many people do not bother to maintain their normal blood pressure levels^2^. Among the main non-medicinal recommendations that should be followed, there are healthy eating, physical activity, reduction of sodium and alcohol consumption, and non-consumption of tobacco^3^.

In Brazil, the units of Family Health Strategie (FHS) lead public health policies and actions with education, promotion, prevention, recovery, rehabilitation, and maintenance of community health^4^. The members of FHS health teams provide primary care for a defined number of families in an attached territory^5^. Organizing their actions and work plan, each FHS arrange the monitoring of patients with AH through periodic appointments, scheduled with a medical doctor or a nurse. These visits include the measurements of weight and blood pressure, along with treatment guidelines, and prescription of medications to promote the monitoring and evaluation of the treatment’s progress^6^.

The use of communication technologies becomes appropriate for the monitoring of patients with AH, since there is a need for solutions that improve the quality of care, which may also contribute to the coverage of treatment and educational actions. Among these technologies, there are mobile health applications (m-Health), the practice of medicine supported by mobile devices^7^. When applied to hypertension treatment, these applications are promising in leading to a significant decrease in blood pressure levels, as well as an effective self-management tool of the disease^8–10^ and improving patients lifestyle^11^.

In the m-Health scenario, Cechetti *et al*.^12^ developed the first version of the e-Lifestyle app and evaluated only patient engagement with the app, which aims to promote healthy habits in AH patients. This app includes features for logging and monitoring measurements of health conditions and habits, such as blood pressure, anthropometric measurements, sleep, and mood. Later, Biduski *et al*.^13^ evaluated the long-term user experience of patients using the app for 3 months, including aspects of the evolution between before, during and after usage. The project was then selected by the Ministry of Health of Brazil for its further development within the National Health System. Therefore, the purpose of this study was to evaluate the feasibility of the approach with a view on preparing the protocol for the definitive trial that will verify the effects of using the app on the health conditions of patients with AH in the public health network.

## Methods

**Figure 1:**
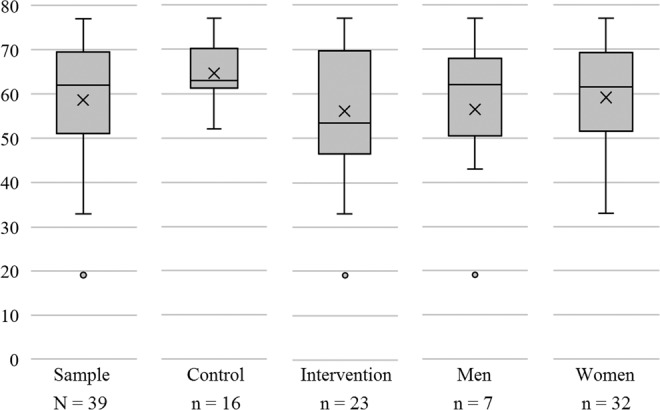
Age of participants in years.

We conducted a non-randomized, controlled, non-blind clinical trial comparing the use of an m-Health app with conventional AH monitoring over 3 months. A total of 45 volunteer participants (of whom 39 completed the study) were recruited, adults and elderly, men and women aged 19 to 77 years (Fig. 1) diagnosed with HA, ongoing medical treatment at FHS in the city of Passo Fundo, Rio Grande do Sul, Brazil. This was a pilot study used to verify the feasibility of the methods and the potential outcomes for the development of an official health innovation project supported by the Ministry of Health of Brazil and the National Council of Scientific and Technological Development of Brazil – CNPq. The results of this study were used to verify the feasibility and specify the characteristics of the definitive clinical trial, already published in its protocol^14^.

This study was conducted according to the guidelines laid down in the Declaration of Helsinki. The local ethics committee of the University of Passo Fundo, under opinion number 1.890.882, approved all procedures involving human subjects/patients. Written informed consent was obtained from all subjects/patients. The trial was registered on the Brazilian Registry of Clinical Trials on March 26, 2019, code RBR-2rkkgn, WHO Universal Trial Number U1111-1230-7550, available at http://www.ensaiosclinicos.gov.br/rg/RBR-2rkkgn/.

The criteria for participation in the research were:

- currently ongoing medical monitoring and follow-up regarding AH treatment;
- have proven cognitive ability in the MMSE psychometric test^15^;
- be able to measure blood pressure periodically;
- be familiar with the use of smartphone apps (for Intervention Group);
- have a smartphone with Android Operating System version 5 or higher (for Intervention Group);
- have Internet access on a smartphone through Wi-Fi or mobile data (for Intervention Group).

The data collection of this study occurred in three moments: pre-intervention, intervention, and post-intervention.

### Pre-intervention

After checking the inclusion criteria and applying the MMSE psychometric test^15^, we collected the following data before the intervention with all participants:

I. Characterization questionnaire, with sociodemographic variables of gender, age, marital status, education, profession, and income. In addition, each participant was asked if he/she understood the meaning of the blood pressure measurement, how often he/she checked his blood pressure, and whether he/she recorded monitoring measurements frequently.

II. Last week’s Food Frequency Questionnaire (FFQ), according to the model of the Food and Nutrition Surveillance System - SISVAN, prepared by the Ministry of Health in Brazil16.

III. Appraisal of Self-Care Agency Scale-ASA-A17.

IV. Blood tests of hemogram, creatinine, uric acid, sodium, potassium, total cholesterol, HDL, triglycerides, and glycemia. A trained professional from our university laboratory collected the blood samples.

V. Measurement of systolic blood pressure and diastolic blood pressure.

The allocation of patients in each group was based on the eligibility criteria. For instance, if the patient met all the criteria but did not have a mobile phone compatible with the app, then he or she would be allocated to the Control Group. If the patient had a mobile phone compatible with the app, he or she would be allocated to the Intervention Group.

### Intervention

The sample was stratified into two groups. The Intervention Group (IG) had 23 participants who completed the intervention using the e-Lifestyle app. The Control Group (CG) had 16 participants and did not have intervention with the app during the study. Blood pressure measurements from IG were read through the data registered on the application, which could be followed by the health professional via the web. For the GC, the measurements were recorded on paper and collected at each meeting. All participants were taking hypertension drugs such as hydrochlorothiazide, losartan, captopril, enalapril or atenolol. During the intervention, no dosage or drug was changed; therefore, the participants continued with their usual treatment.

Usually, health education workshops are one of the FHS strategies, which are conducted in support groups for patients with chronic diseases on a monthly or less frequent basis. For this study, workshops were conducted more frequently. Every 15 days during the intervention, participants from both groups attended scheduled workshops at the FHS health unit to receive nutritional guidance and information about health habits from dietitians and nurses. The workshops took place in the form of lectures and talks, always performed by the same professionals in a standardized way. At those meetings, the participants also had their blood pressure values and other anthropometric data measured.

#### Control group

We instructed CG participants to measure their blood pressure during the 3 months of the intervention period, storing the information as they usually did before, according to their conventional monitoring. Thus, all participants made notes on paper and brought us in the meetings. On average, participants measured blood pressure once a day during the period.

#### Intervention group

**Figure 2:**
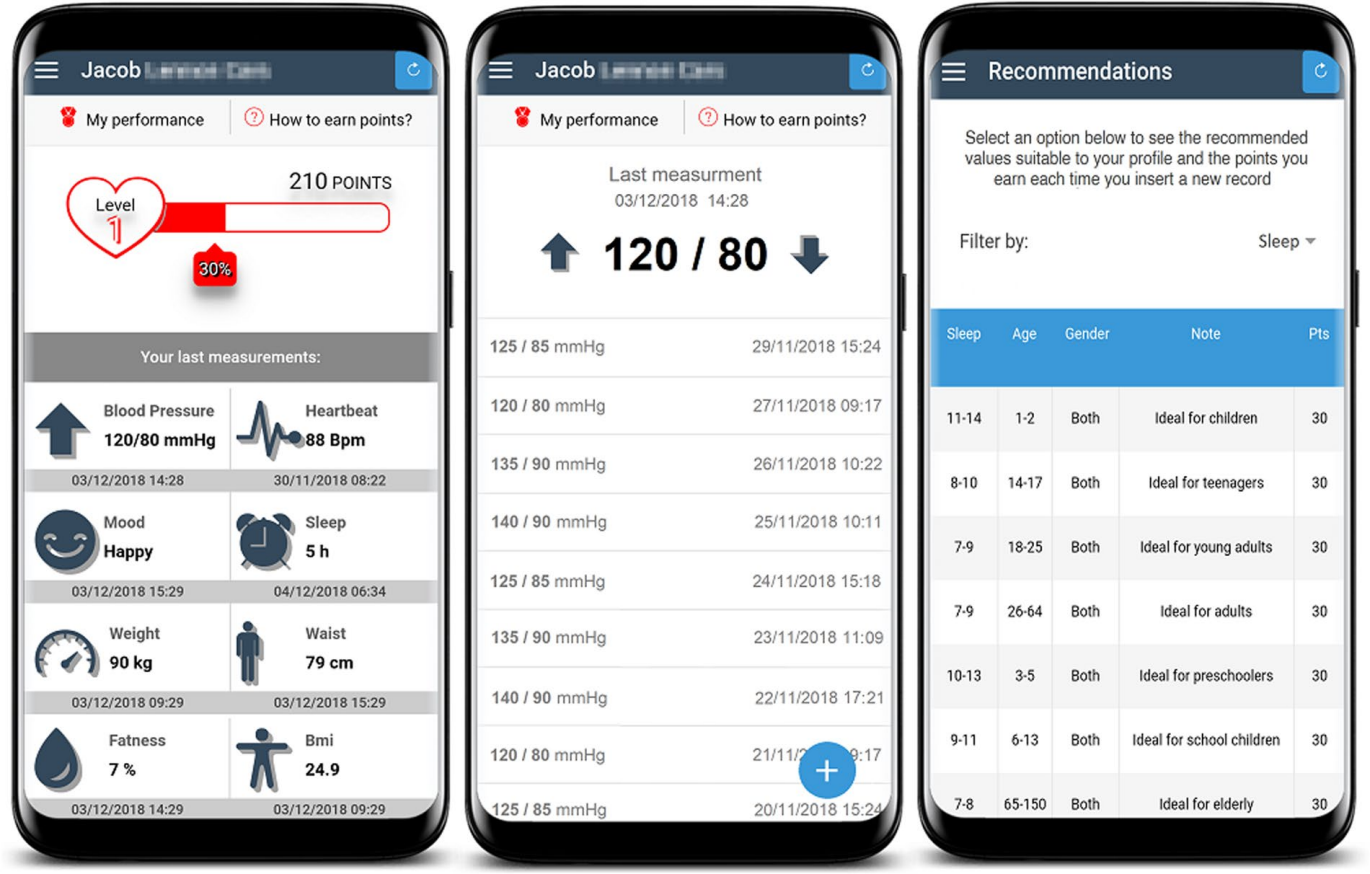
Screenshots of the e-Lifestyle app.

First, IG participants had the e-Lifestyle app (Fig. 2) installed on their smartphones. After that, we created a user for each participant and instructed him or her on how to use the app. Participants were asked to record data such as blood pressure and other measurements on the app. We helped participants who had difficulties with the app, giving feedback and more detailed instructions.

The IG participants used the e-Lifestyle app for 3 months. According to Neumann *et al*.^16^, this period is suitable to accomplish possible significant changes in blood pressure levels through weight loss and balanced diet, along with other healthy habits. The app has elements that aim to engage patients in their own treatment, as demonstrated by Cechetti *et al*.^12^. It allows the recording of variables related to blood pressure, weight, waist circumference, height, sleep, mood, and the practice of physical activities. Other features include risk assessment, recommendations based on reference values, alerts and reminders. The app has a dashboard designed so that the patient has on one screen the summary of their most important health conditions. In-app patient data is stored in a computational cloud for integration with a web dashboard, which allows a FHS’ healthcare professional to remotely monitor the patient.

### Post intervention

After 3 months of intervention, we collected the following data again:

I. Last week’s Food Frequency Questionnaire (FFQ), according to the model of the Food and Nutrition Surveillance System - SISVAN, prepared by the Ministry of Health in Brazil^17^.

II. Appraisal of Self-Care Agency Scale-ASA-A^18^.

III. Blood tests of hemogram, creatinine, uric acid, sodium, potassium, total cholesterol, HDL, triglycerides, and glycemia. A trained professional from our university laboratory collected the blood samples using the same technique as in the pre-intervention.

IV. Measurement of systolic blood pressure and diastolic blood pressure.

### Statistical analysis

Quantitative data were analyzed using the statistical package SPSS 22.0 (IBM Corporation). We used the Wilcoxon (paired) or Mann Whitney (independent) test to compare the means, since the variables did not show normal distribution by the Kolmogorov-Smirnov and Shapiro-Wilk test. Thus, the Wilcoxon test was used for the comparison between the pre- and post-intervention values of the blood tests, FFQ, and ASA-A. For the ASA-A scores, we also applied the Mann Whitney test to compare the self-care score between the groups before and after the intervention. The same test was used to compare diastolic and systolic blood pressure measurements between the pre and post-intervention groups. A significance level of 5% was considered for all analyzes.

To verify the factors associated with the app use, we performed logistic regression of the data collected pre and post intervention. The dependent variable considered the base category “did not use the app” (CG) and the interest category “used the app” (IG). We related the dependent variable with the independent variables of interest, which are: gender (categorized), age (continuous), level of education (categorized), pre/post ASA-A self-care score (continuous), pre/post systolic blood pressure (continuous), and pre/post diastolic blood pressure (continuous).

## Results

**Figure 3:**
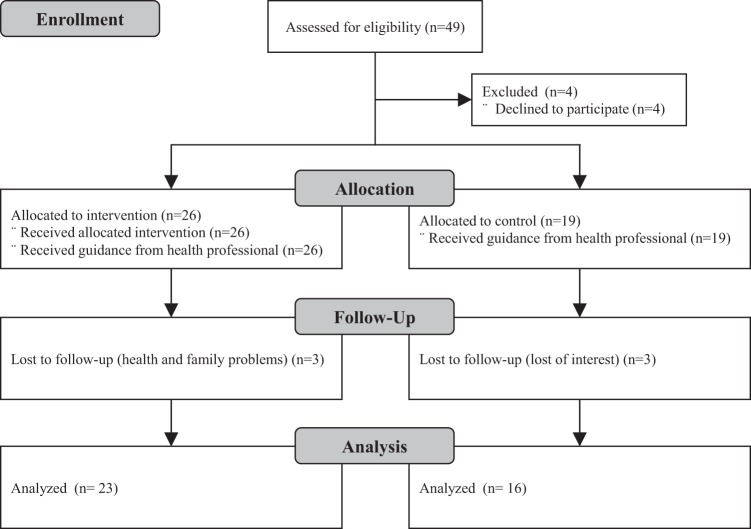
Consort study flow diagram.

During the evaluation, 45 participants were recruited. However, 6 dropped out before the end of the study (Fig. 3). Among those, 3 were participants that belonged to IG, who used the app for only 2 weeks. According to them, the reason for abandonment was related to family and health problems. The other 3 participants belonged to CG and dropped out because they had no further interest in participating.

Table 1 summarizes the sociodemographic characteristics of the sample. The majority were female, both in the intervention group (87%) and in the control group (75%), with a prevalence of widows and married women. About the level of education, 61.6% of participants affirmed they had primary education. The same level of education was reported by 52.2% of the participants in the intervention group, and by 75.1% in the control group. To verify the homogeneity of the educational variable between the two groups, we applied Fisher’s exact test, classifying educational stages in two categories, namely: (a) until incomplete high school and (b) high school or higher education. Results indicated there was no significant difference (p = 0.158) between the control group (a = 48.1%, b = 25%) and intervention group (a = 51.9%, b = 75%), data not shown in the table.

**Table 1:** Demographic and socioeconomic characteristics of adults and elderly patients attended at basic health units, Passo Fundo, RS, Brazil, 2019 (N = 39).

Variable	Category	Whole sample		Intervention		Control	
		N	%	n	%	n	%
Gender	Male	7	17.9	3	13.0	4	25.0
	Female	32	82.1	20	87.0	12	75.0
Marital status	Single	4	10.3	3	13.0	1	6.3
	Married	17	43.6	12	52.5	5	31.3
	Widow(er)	18	46.2	8	34.8	10	62.5
Schooling	Incomplete elementary school	15	38.5	8	34.8	7	43.8
	Complete elementary school	9	23.1	4	17.4	5	31.3
	Incomplete high school	3	7.7	2	8.7	1	6.3
	Complete high school	11	28.2	8	34.8	3	18.8
	Higher education	1	2.6	1	4.3	-	-
Income	Have income	31	79.5	18	78.3	13	81.3
	Have no income	8	20.5	5	21.7	3	18.8
Understood the meaning of blood pressure measurement	No	2	5.1	1	4.3	1	6.3
	Yes	37	94.9	22	95.7	15	93.8
Frequency of measuring blood pressure	Once a day	4	10.3	2	8.7	2	12.5
	≥ twice a day	2	5.1	—	—	2	12.5
	Twice a week	12	30.8	8	34.8	4	25.0
	≥ twice a week	4	10.3	2	8.7	2	12.5
	Once a month	17	43.6	11	47.8	6	37.5
Whether write down the blood pressure measurements	No	25	62.2	14	60.9	9	64.3
	Yes	10	27.0	6	26.1	4	28.6
	Sometimes	4	10.8	3	13.0	1	7.1
Occupation	Unemployed	2	5.1	2	8.7	—	—
	Housewife	10	25.6	6	26.1	4	25.0
	Worker	19	48.7	12	52.2	7	43.8
	Retired	7	17.9	2	8.7	5	31.3
	Student	1	2.6	1	4.3	—	—

Regarding income, data showed that about 80% of the participants had some kind of income, independent of the group. The sample was composed mostly of workers and retirees. When participants were asked about understanding the meaning of blood pressure measurement, most said they did understand it. As for the frequency of blood pressure measurement, most said they measured only once a month and did not record their data for further evaluation.

Comparing the results of blood tests before and after the intervention (Table 2), IG lowered values of glucose (p = 0.009), total cholesterol (p = 0.008), HDL cholesterol (p = 0.033) and non-HDL cholesterol (p = 0.014). In CG, total cholesterol levels (p = 0.035) and non-HDL cholesterol (p = 0.023) were reduced.

**Table 2:** Description and comparison of clinical exams before and after the intervention (values 1 and 2) of adults and elderly patients attended at basic health units, Passo Fundo, RS, Brazil, 2019 (N = 39).

Blood test	Intervention Group			*P*	Control Group			*P*
	Mean	SD	Median		Mean	SD	Median	
Blood glucose 1	172.84	131.54	119.80	**0.009**	122.91	58.95	107.06	0.683
Blood glucose 2	140.75	64.40	120.30	*	117.29	46.97	94.23	
Creatinine 1	0.8126	0.1877	0.8000	0.643	0.9175	0.2352	0.9050	0.784
Creatinine 2	0.8243	0.2140	0.8000		0.8794	0.2598	0.8350	
Sodium 1	137.34	1.55	137.00	0.275	137.81	2.42	137.50	0.552
Sodium 2	137.65	1.72	138.00		138.06	1.69	138.00	
Potassium 1	3.93	0.3069	3.90	0.419	4.10	0.3855	4.10	0.245
Potassium 2	4.01	0.3900	4.00		3.93	0.3995	3.80	
Total cholesterol 1	194.55	38.43	200.00	**0.008**	190.31	37.38	186.50	**0.035**
Total cholesterol 2	175.90	34.13	180.90	*	177.06	37.67	172.60	*
HDL cholesterol 1	38.11	10.86	35.10	**0.033**	41.20	12.04	38.65	0.196
HDL cholesterol 2	35.20	8.93	32.60	*	38.90	10.00	39.10	
Non-HDL cholesterol 1	157.09	39.64	161.98	**0.014**	148.40	37.34	143.93	**0.023**
Non-HDL cholesterol 2	139.19	32.52	123.71	*	136.06	38.40	119.57	*
Triglycerides 1	203.05	143.09	160.67	0.970	165.46	120.12	141.02	0.463
Triglycerides 2	186.82	71.80	198.35		166.17	81.99	139.77	
Uric acid 1	4.10	0.77	4.09	0.809	4.02	0.78	4.10	0.695
Uric acid 2	4.09	0.84	3.97		4.10	1.01	3.98	

Regarding food consumption (Table 3), IG increased the frequency of salads consumption and reduced the consumption of processed meat products, cookies or sweets, and sugary drinks (p < 0.05). The CG increased the consumption of salads and fruits and reduced the consumption of sugary drinks (p < 0.05).

**Table 3:** Comparison of the frequency of food consumption, in serves per week considering the last week, before and after the intervention (values 1 and 2), of adults and elderly patients attended at basic health units, Passo Fundo, RS, Brazil, 2019 (N = 39).

Food item	Intervention Group			*P*	Control Group			*P*
	Mean	SD	Median		Mean	SD	Median	
Salad 1	3.83	2.79	3.0	**0.006**	4.25	2.24	4.0	**0.020**
Salad 2	5.7	1.92	7.0	*	6.0	1.86	7.0	*
Vegetable 1	2.22	2.39	2.0	0.063	2.75	1.98	2.0	0.165
Vegetable 2	3.17	2.38	3.0		3.63	1.89	3.0	
Fruits 1	4.22	2.58	3.0	0.089	2.94	2.77	3.0	**0.010**
Fruits 2	5.0	2.52	7.0		4.88	2.34	5.0	*
Beans 1	5.0	2.56	7.0	0.811	5.63	2.03	7.0	0.142
Beans 2	5.04	2.50	6.0		6.44	1.21	7.0	
Milk 1	4.87	2.82	7.0	0.721	4.81	2.81	6.0	0.235
Milk 2	4.70	3.02	7.0		4.13	2.85	4.5	
Fried foods 1	1.83	2.29	1.0	0.089	1.06	1.24	1.0	0.261
Fried foods 2	1.0	1.60	1.0		1.88	2.39	1.0	
Processed meat products 1	2.61	2.66	2.0	**0.018**	2.13	1.96	2.0	0.297
Processed meat products 2	1.04	1.55	1.0	*	1.63	2.31	1.0	
Savory biscuits 1	2.52	2.68	2.0	0.247	2.38	2.45	1.5	0.098
Savory biscuits 2	1.78	2.54	1.0		1.38	2.13	1.0	
Cookies or sweets 1	2.17	2.59	1.0	**0.002**	2.0	1.86	2.0	0.081
Cookies or sweets 2	0.61	1.56	0	*	1.0	1.83	0	
Sugary drinks 1	1.87	2.18	1.0	**0.010**	1.31	1.82	1.0	**0.032**
Sugary drinks 2	1.04	1.75	0	*	0.44	0.63	0	*

Self-care data (Table 4), showed no significant difference between the groups at the beginning and at the end of the study (beginning p = 0.053; end p = 0.333). However, results indicate there was an improvement in the self-care score for both groups.

**Table 4:** Comparison of ASA-A self-care score before and after the intervention of adults and elderly patients attended at basic health units, Passo Fundo, RS, Brazil, 2019 (N = 39).

ASA-A Scale	Intervention Group			*P*	Control Group			*P*
	Mean	SD	Median		Mean	SD	Median	
Self-care score 1	96.52	12.89	99.0	**≤0.001**	99.13	16.48	100.5	**0.002**
Self-care score 2	106.87	9.32	108.0	*	111.25	9.77	114.5	*

In the comparison of blood pressure measurements (Table 5), there was no significant difference between the groups at the beginning and at the end of the study (systolic blood pressure: beginning p = 0.301, end p = 0,314; diastolic blood pressure: beginning p = 0.525, end p = 0.595). However, in the IG, diastolic blood pressure values increased, and systolic blood pressure values decreased (p < 0.05) towards more adequate values, which shows improvement. No significant difference was observed at the beginning and at the end of the study in CG.

**Table 5:** Comparison of diastolic and systolic blood pressure before and after the intervention (values 1 and 2).

Blood pressure	Intervention Group			*P*	Control Group			*P*
	Mean	SD	Median		Mean	SD	Median	
Systolic 1	133.7	11.4	130.0	**≤0.001**	134.0	10.5	130.0	0.617
Systolic 2	127.9	11.3	120.0	*	132.6	12.2	140.0	
Diastolic 1	72.3	10.2	80.0	**0.004**	75.3	8.3	80.0	0.064
Diastolic 2	83.9	7.2	90.0	*	82.7	7.0	80.0	

In the pre-intervention data analysis using the multiple logistic regression model, no variables were associated with app use (gender, age, level of education, diastolic blood pressure, systolic blood pressure, ASA-A self-care score). In the analysis of post-intervention data using the multiple logistic regression model, the only variable associated with the use of the application was the post-intervention systolic blood pressure (OR = 0.883; 95% CI: 0.781–0.998; p = 0.046), adjusted for other variables (gender, age, level of education, diastolic blood pressure, systolic blood pressure, ASA-A self-care score).

Overall, there were beneficial effects verified in the several variables measured, even in the control group, which we did not originally foresee. We realize that intensifying health education workshops had substantial prospects in the context of the FHS and this is worthy of being investigated in future studies. However, it restricts our main objective, which is to verify what is exclusively app’s effects. Hence, from the point of view of feasibility, we concluded from this pilot that the definitive trial cannot provide additional workshops. Besides, the sample size will be recalculated and substantiated with a propensity score that will consider the effects pre-verified in this pilot towards a better balance. Meanwhile, the evaluation of many outcome variables becomes a complex task for correlating all the findings, hindering the establishment of solid conclusions. Therefore, the definitive trial will focus on systolic and diastolic blood pressure as the primary outcome, considering that these are the main reference parameter in the AH treatment. Nevertheless, secondary studies should address the other variables tested in this pilot.

## Discussion

In relation to the demographic and socioeconomic characteristics, the profile found in this research resembled surveys performed in similar scenarios, concluding that the majority of participants are workers or retirees living in the area covered by the FHS^6^. Socioeconomic status and education appear as risk factors for chronic noncommunicable diseases and cardiovascular diseases^6^. Health professionals should encourage measures that impact lifestyle, but their adoption falls to the patient’s understanding of his/her own problem, to the motivations and conditions associated with the treatment. In this sense, income and education, as social determinants of health, are closely related to hypertension^19^. During the study, all patients had regular access to a doctor at the FHS, following their exams and clinical picture. Furthermore, participants from both groups had the assistance of a collaborative team of professionals. However, these results do not allow generalization and can only be interpreted from the results of the definitive clinical trial.

Regarding the blood tests performed, there was a significant change in the glucose levels in the IG. This attribution is mainly due to the comorbidity of AH and Diabetes Mellitus (DM), common in adult and elderly populations^20^. Total cholesterol decreased in both groups, with a more significant improvement for IG. Non-HDL cholesterol had lower levels in both groups, especially the significant improvement in IG, highlighting the positive effects of the app. The HDL cholesterol had a significant difference only for IG. The adequate diet associated with the practice of regular physical exercises may have contributed to the results found, probably motivated by the use of the app with its features of weight and BMI monitoring. Overall, cholesterol showed significant improvements, unlike triglycerides, which require a longer period of dietary reeducation and healthy habits to achieve more considerable changes^21^.

Sarkar *et al*.^22^ report that the lipid profile of patients with hypertension is commonly worse than patients with normal blood pressure. The improvement in the lipid profile is an remarkable result, considering that more than half of the decrease in cardiovascular mortality has been attributed to changes in risk factors in the population, especially the lower levels of cholesterol, blood pressure and smoking^23^. Similar studies — addressing body weight, waist measurement, blood pressure, and lipid profile — showed improvements in lipid levels with the use health information technology^24,25^.

The increase in the consumption of salads was evidenced in both groups. This result and other positive changes in eating habits may have occurred because the sample was exposed to the dietitian’s guidance during the intervention period. However, it is not ruled out that the results could be enhanced by the use of the app in the IG, nor that the app could produce effects without being associated with the guidance from dietitians. Whelton^26^ details this as one of the outstanding improvements provided by strategies such as empowering patients through mechanisms such as education and self-monitoring of blood pressure, including the use of technology to provide reminders and encouragement. Healthy dietary behavior, as a modifiable factor of cardiovascular disease, is crucial for the prevention and early intervention in AH^27^.

Both groups decreased sugary drinks consumption. In addition, the IG exclusively significantly decreased in the consumption of processed meat products (p = 0.018) and filled biscuits (p = 0.002). Those are industrialized foods, rich in sodium and saturated fats, which may raise cholesterol levels that influence AH^28^. These are also substantial improvements, given the difficulties encountered by patients in maintaining a healthy diet. The official food recommendations in Brazil define that the food base should be fresh, and ultra-processed foods should be avoided^29–32^.

The self-care scale assessment (ASA-A) revealed improvements for both groups, probably due to FHS monitoring with regular feedback and health care. This suggests that the app was a kind of incentive for care, but also that the health education workshops provided had a positive influence on these values. These results also underline the valuable role of health education provided by professionals in the context of FHS. The app’s features of reminders, sleep, mood, physical activity, and food information become attractive for a healthier lifestyle and improvement on several factors of self-care and self-management^33^. Because the interactive support tool encourages patient’s self-registration of variables related to health and provides feedback on changes, patient engagement is augmented^34,35^. Registering daily health data on an app can motivate the patient to create a routine, paying more attention to their health by improving adherence to treatment^13,36^. In this sense, we can assume that using the app has positively influenced the patient’s commitment to medication intake and, consequently, has influenced with better outcomes in the IG.

Finally, the most important result of this study was the improvement in systolic and diastolic pressure levels observed in IG. Both values shifted towards healthier reference standards (120 mmHg systolic, 80 diastolic mmHg) defined by Brazilian guidelines^37^. Therefore, the present study corroborates similar results in the literature^38–41^, reaffirming the many benefits of using m-Health technology to treat AH.

Giving feedback and orientation to the population is one of the strategies organized by the FHS to promote health, which includes guidance on healthy eating, regular physical activity, group activities with seniors, workshops and lectures on hypertension and diabetes^42^. With the FHS actions, patients participate as actors in the construction of knowledge and practices that aim to favor health conditions and, consequently, patients can achieve independence in the decision-making process, with new possibilities to improve lifestyle^43^. In the context of FHS and primary care, digital technologies can assuage health care discrepancy, enabling the provision of integrated information on disease management, especially hypertension^44–46^. Using m-Health technologies represents an attractive option to improve health behaviors and outcomes given the widespread use of these technologies^47–49^. Feedback, workshops, and primary care offered by the FHS can be enhanced by using m-Health. All the positive consequences reassure the advantages of using technology and its relevant impact on the public health context by lowering costs and improving the quality of care^50–52^.

## Limitations

There are some limitations in the study; among them, the difficulty of adherence to the use of the app. Over the 3 months, users needed to be encouraged with workshops and live feedback so they could handle the app’s features. Consequently, withdrawals and the lack of knowledge about research participation made it difficult to recruit patients for the study. Although the workshops showed good results in adherence and continuance in the study, they may have interfered with the outcomes, making the interpretation of the findings difficult. Regarding the study design, non-randomization may have had a bias influence on the findings, as well as the difference of 7 participants between groups. However, the two groups were similar concerning the demographic and socioeconomic aspects, minimizing the possible biases. Besides, the impossibility of blinding health professionals and interviewers may have contributed to the information bias. Nevertheless, all those involved in the research underwent training to lessen the possibility of bias.

## Conclusions

In this pilot study, we aimed to preliminarily validate the intervention and assessment method and clinical effects of the e-Lifestyle app. The version used does not yet include all the factors necessary for the treatment of AH. Even so, the outcomes are encouraging: there is evidence that using an app can have beneficial effects on the health conditions of patients with AH. The use of technologies combined with health information is a positive development that can contribute to the therapeutic scheme of patients with hypertension, providing greater adherence to treatment, healthier habits, and better health conditions. Improvements were identified in both groups due to the health education workshops. However, the intervention group, which used the m-Health app, performed better. Even though the app combined with the workshops of FHS has the potential for more positive outcomes, this needs to be confirmed in further studies with a suitable technique. From the results, we could understand the benefit and association of each app feature with health outcomes. We concluded that the first goal, to be met in the definitive clinical trial, is to verify what is exclusively the effect of the app on FHS patients. With this determination, other studies will then be able to verify the best way of delivering or associating app intervention. Accordingly, we better adjusted the intervention technique, the expected outcomes, and the assessment frameworks to create the definitive trial’s protocol^14^ using a new improved and comprehensive app’s version.

## Data Availability

The data that support the findings of this study are available from the corresponding author upon reasonable request.
